# Phalangeal bone anomalies in the European common toad *Bufo bufo* from polluted environments

**DOI:** 10.1007/s11356-016-7297-6

**Published:** 2016-08-18

**Authors:** Mikołaj Kaczmarski, Krzysztof Kolenda, Beata Rozenblut-Kościsty, Wioletta Sośnicka

**Affiliations:** 1 Institute of Zoology, Poznan University of Life Sciences, Wojska Polskiego 71 C, 60-625 Poznań, Poland; 2 Department of Vertebrate Biology and Conservation, University of Wroclaw, Sienkiewicza 21, 50-335 Wrocław, Poland

**Keywords:** Amphibians, Phalangeal bone, Poland, Pollution, Roadkilling, Skeletochronology, Urban area

## Abstract

Every spring, many of amphibians are killed by motor vehicles on roads. These road-killed animals can be used as valuable material for non-invasive studies showing the effect of environmental pollution on amphibian populations. The aims of our research were to check whether the phalanges of road-killed toads may be useful as material for histological analysis, and whether various degrees of human impact influence the level in bone abnormalities in the common toad. We also examined whether the sex and age structure of toads can differ significantly depending in the different sites. We chose three toad breeding sites where road-killed individuals had been observed: near the centre of a city, the outskirts of a city, and a rural site. We collected dead individuals during spring migration in 2013. The sex of each individual was determined and the toes were used to determine age using the skeletochronology method. While performing age estimates, we looked for abnormalities in relation to normal bone tissue structure. In urban site, females dominate males (sex ratio 2.6:1), but in populations from rural and semi-urban sites, sex ratio was reverse (1:2.2 and 1:1.4, respectively). However, we did not find any significant differences between age structure of all populations (average age of each population: approximately 4 years). We observed abnormalities in more than 80 % of all toads from the city, compared to approximately 20 % from the rural and semi-urban sites. In particular, we found hypertrophic bone cells, misaligned intercellular substance, and irregular outer edges of bones. We suggest that these malformations are caused by different pollution, e.g. with heavy metals.

## Introduction

Current knowledge widely indicates a direct relationship between anthropogenic pressures and the deteriorating environmental state and/or global extinction of amphibian populations (Blaustein and Johanson 2003; Collins and Storfer 2003; Stuart et al. 2005). Negative impacts include direct killing (Santos et al. 2015), destruction of habitats (Denoël 2012), and poisoning of the environment (Egea-Serrano et al. 2012). In Europe, many amphibian populations are threatened (Temple and Cox 2009; Bonk and Pabijan 2010; Denoël 2012; Kaczmarek et al. 2015). In terms of the challenge of preventing these negative processes, it is necessary to develop techniques for detecting disorders in natural processes and changes in the environment and to study their impact on amphibians (Egea-Serrano et al. 2012). Research on the effects of chemicals on the development and survival of tadpoles have been carried out in laboratories around the world (Skei and Dolmen 2006; Hayes et al. 2010b; Distel and Boone 2011; Orton and Routledge 2011). Impact of pollution on amphibians, encompassing pathology and histological analysis, is usually based on captured living adults. However, these studies are controversial, raising ethical issues, as they make it necessary to capture and kill adult individuals (Söderman et al. 2007; Dmowski et al. 2015). Therefore, this kind of survey is not as frequent as research on tadpoles or laboratory research. Considering that over 30 % of amphibian species are threatened with extinction, the attention of researchers is being directed toward non-invasive methods (Simon et al. 2009, 2012; Orton et al. 2014). The road mortality of vertebrates offers great opportunities for obtaining material for further research (D’Amico et al. 2015; Santos et al. 2015), including studies on bioindication (Simon et al. 2012). For instance, road-killed toads were used in research on heavy metals absorbed by bone tissues (Simon et al. 2009, 2012).

The accumulation of contamination, e.g. heavy metals or biogenic compounds, in amphibians’ body organs and tissues, and its impact on their physiology and behaviour are well known (Huey and Beitinger 1980; Lefcort et al. 1997; Rouse et al. 1999; Greulich and Pflugmacher 2003; Dobrovoljc et al. 2012; Egea-Serrano et al. 2012). Unfortunately, there is still little knowledge about the effect of these contaminants on life history traits and recruitment of amphibians (Rowe et al. 2001; Hayes et al. 2010a). Some studies exhibit an enhanced association between the occurrence of body malformation (e.g. missing or extra limbs, limb deformities) in amphibians and the increasing land use in human-associated (Blaustein and Johanson 2003; Taylor et al. 2005; but see Ballengée and Sessions 2009).

Common species with wide distributions are useful for research, as it is relatively easy to obtain sufficiently large samples and to conduct research on a large spatial scale (Sparks et al. 2007). In Europe, including Poland, one of the most widespread species, but also one of the most vulnerable to road mortality, is the common toad *Bufo bufo* (Głowaciński and Rafiński 2003; Elzanowski et al. 2009; Temple and Cox 2009)*.* Moreover, this species is used with increasingly frequency in attempts to assess the state of environmental pollution (Orton and Routledge 2011; Dobrovoljc et al. 2012; Simon et al. 2012; Orton et al. 2014; Dmowski et al. 2015; Pickford et al. 2015). Research in urban areas has shown that the number of sites occupied by amphibians, including common toads, has decreased slightly (Budzik et al. 2013; Kaczmarek et al. 2015). A similar situation is observed in less-urbanised habitats (Orłowski 2007; Beebee 2014). This may be caused not only by road mortality and habitat loss but also by high levels of water and soil pollution which penetrates into amphibians’ bodies and contributes to increasing their mortality rates and reducing their level of fitness. *B. bufo* exhibits a broad range of environmental tolerances and therefore may be exposed to the influence of various substances that disrupt its development, particularly in anthropogenic environments (Dmowski et al. 2015; Pickford et al. 2015). To the best of our knowledge, there are no data which demonstrate the appearance of pathological changes in the bones of amphibians inhabiting a polluted environments.

We investigated two main aims of this study: whether the phalanges of road-killed toads may be useful as material for histological analysis, and whether various degrees of human impact (urban, semi-urban and rural localities) influence the level in bone abnormalities in the common toad. Our hypothesis assumes that toads occurring at an urban site are more prone to the formation of anomalies in bone tissue than those from rural localities. We also examined whether the sex and age structure of toads can differ significantly depending in the different sites.

## Material and methods

**Fig. 1:**
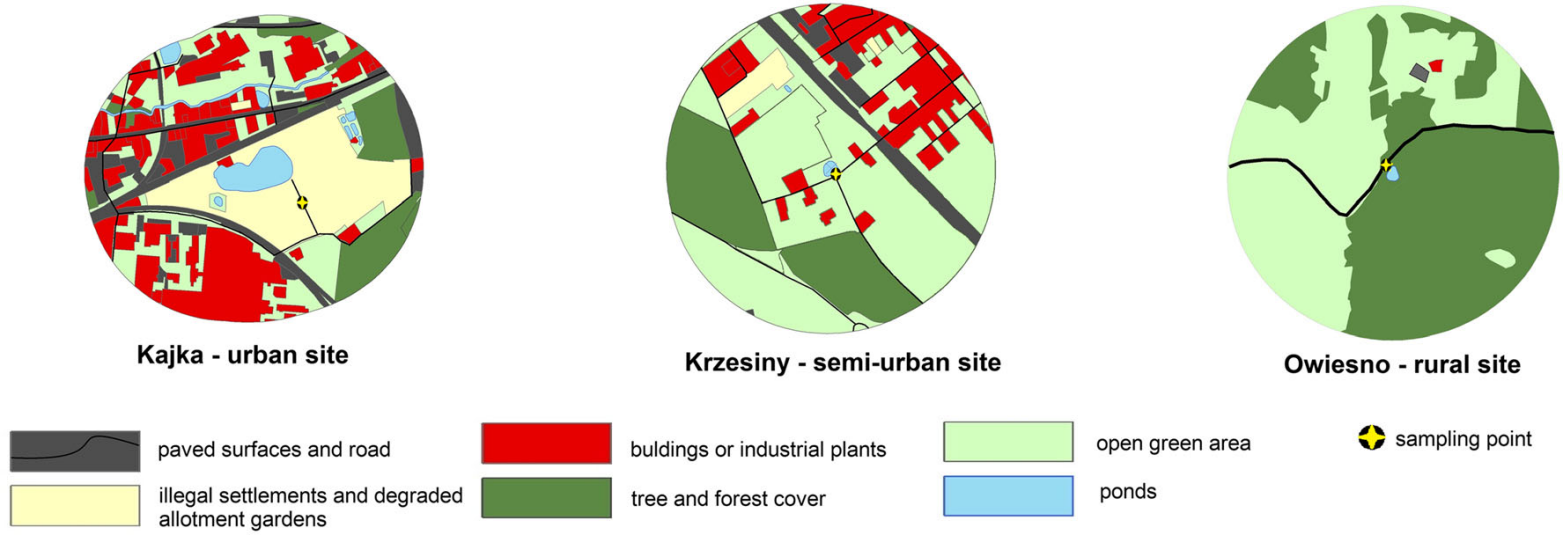
Land covers of 1 km buffers used to quantify degrees of urbanisation around breeding side near sampling points. Land covers are tree and forest cover (*dark green*), paved surfaces (*black*), open green area (farmlands, meadows, fallow lands) (*light green*), illegal settlement and degraded allotment gardens (*sand*) and buildings or industrial plants (*red*)

We chose three breeding sites of the common toad that characterised by varied in the degree of urbanisation and pollution. During the site selection, we analysed the use of catchment basin, in particular the ratio between the forest/tree cover with open green area compared to the paved surfaces (parking lots, railroad, road) with buildings or industrial plants (Fig. 1.). We evaluated the characteristics of the surface runoff which is reflected in the accumulation of pollutants. **Site 1.—**the most polluted urban site was ‘Kajka’, situated near the center of the Poznań city (52.2518° N, 16.5910° E). In the immediate vicinity, there are located an illegal settlement and degraded allotment gardens without sanitary sewer lines. Furthermore, there are two railway lines, and on the east, there is a forest area (approximately 0.5 km from the pond). In ‘Kajka’ site, there is a large proportion of industrial plants in the catchment basin. The pond and surrounding settlement are contaminated by factory halls and storage sites located in the vicinity. Presence of heavy metals and petroleum hydrocarbons have been revealed in sediments which pose a threat for aquatic environment (Grabia 2013). Occasionally, mass fish deaths are observed (Sołtysiak 2013). **Site 2.—**semi-urban site was ‘Krzesiny’ located in the southern periphery of the Poznań city (52.3370° N, 16.9795° E). In the nearest vicinity of the pond, there are farmlands, meadows, fallow lands and small forest. Among the anthropogenic structures, we distinguished railways, roads, a military air base and a few houses—a remnant of the old farms. On the east, the railway line forms a barrier between the surrounding area of toads’ breeding site and the area occupied by residential houses—the developed area. There is no direct discharge of water surface to the pond, but in the whole area, there are drains with the system of wells which causes water seepage. Aquatic vegetation is systematically removed every year. **Site 3.**—the rural site, natural vegetation (meadows) and a pond located in the forest, are situated near the Owiesno village (50.6623° N, 16.7034° E). The catchment area is similar to the natural environment with the small watercourse flowing in the nearest vicinity of the breeding site. A slight extensive farm and local road with little car traffic is located within a radius of 1000 m is only. Very clean water in the pond promotes the development of freshwater plants.

As material, we used dead individuals collected from roads situated between their wintering area and breeding ponds (in the immediate vicinity of breeding pond less than 100 m). All toads were picked up during spring migration in March/April 2013. The sex of individuals was determined in the field by the nuptial pad in males and ovaries and eggs in females, as well as by body size and shape. Each toad was put to the single string plastic bag and transported to the laboratory. The age was determined by the skeletochronology method (Rozenblut and Ogielska 2005). Phalange of hind limb were dissected, then soft tissues were removed from bones and clean ones were decalcified in 1:1 mixture of 10 % formic acid and 4 % formalin for 2–4 h. Decalcified phalanges were washed in pure water (four changes, 10 min each). Such prepared bones were stored in 80 % ethanol. One phalange of each toad were embedded in freezing medium (Jung, Nussioch, Germany) in silicon rubber flat embedding moulds (Pelco International, Redding, CA), then sliced into 10-μm thick sections using Leica CM 1900 freezing microtome. Right before age estimation, bone sections were stained in 0.05 % cresyl violet. In order to evaluate the age of each individual, the number of lines of arrested growth (LAG), deposited in periosteal bone during each hibernation (1 LAG = 1 hibernation = 1 year of life), was counted (Castanet and Smirina 1990). Then, any deviations from normal bone tissue structure were searched. Histological analysis was performed using a Carl Zeiss Axiostar 20 microscope.

To estimate differences between age structure of population, we used Kruskal–Wallis H test. We used contingency table and Pearson chi-square test to compare frequency of anomalies occurrence between studied sites. These analyses were made in the statistical software (StatSoft Inc. Statictica ver.12). We also tested frequency of bones abnormalities occurrence between males and females and between years of life using binomial generalised linear model (GLMM) in R. Confidence intervals for prevalence of anomalies (95 % CI) were computed using the Quantitative Parasitology application (Rózsa et al. 2000).

## Results

At the urban site, 208 road-killed toads were collected, with a ratio of females to males of 2.6:1, whereas at the semi-urban site (*N* = 56) the ratio was 1:2.2 and at the rural site (*N* = 41) 1:1.4 (Table 1).

**Table 1:** Sex ratio, average age and percent of toads with bone abnormalities from studied sites

Locality	Sample size	Level of urbanisation	Sex ratio (♀:♂)	Average age	Percent prevalence of anomalies (95 % CI)
Kajka	208	Urban	2.6:1	3.7 ± 0.86	82 (73–89)
Krzesiny	56	Semi-urban	1:2.2	3.8 ± 0.84	23 (13–36)
Owiesno	41	Rural	1:1.4	3.9 ± 0.87	19 (9–35)

**Fig. 2:**
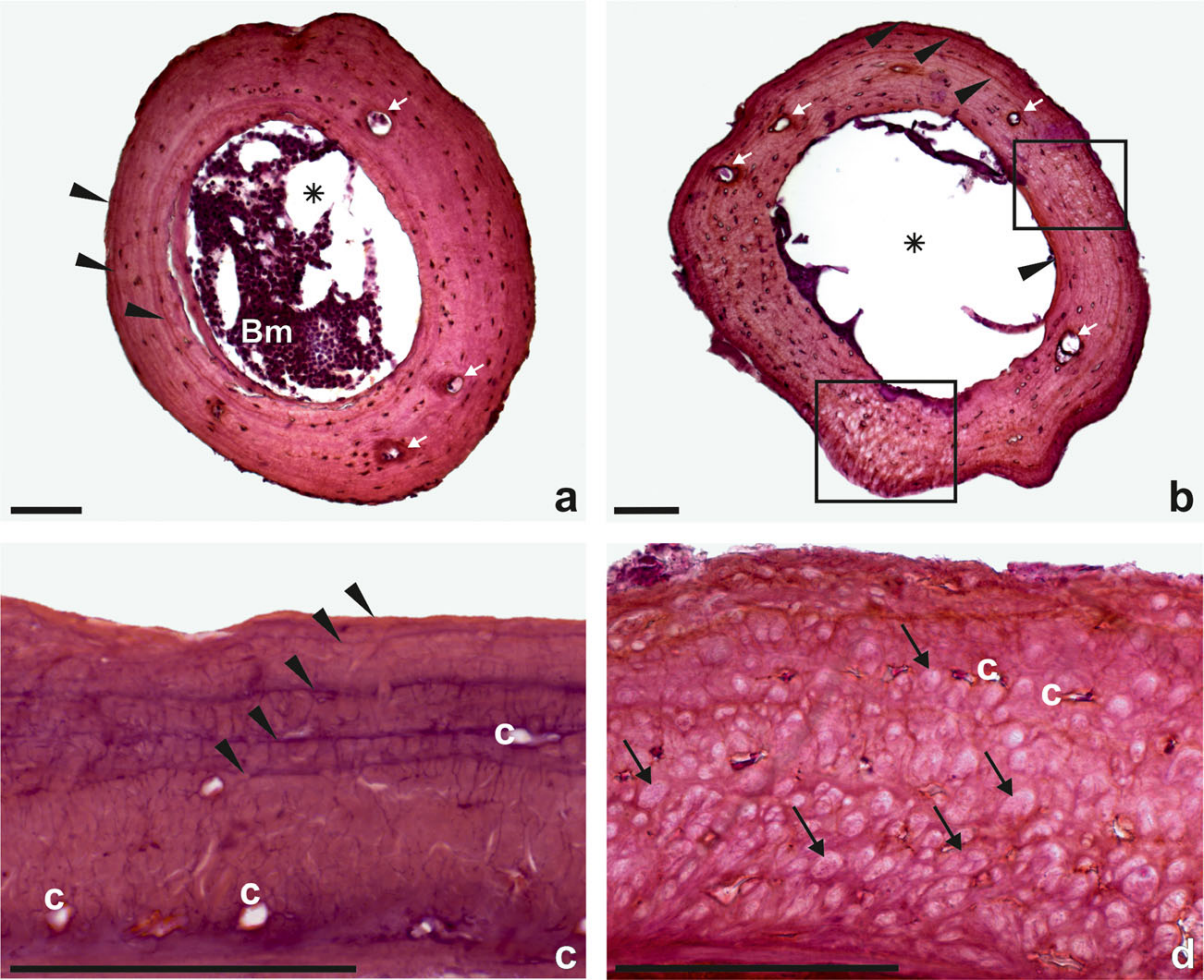
Transverse sections of third phalanx (diaphysis) of toads: after third hibernation—typical (**a**), fourth hibernation—with anomalies (in *black frame*) (**b**). Transverse sections of epiphysis part of toad’s phalanx: after sixth hibernation—typical (**c**) and anomalies in bone tissue (toad of unknown age) (**d**). *Black arrow heads*—line of arrested growth (LAG), (*asterisk*)—bone marrow cavity, *Bm*—bone marrow present in the marrow cavity, *white arrow*—nutrition canal, *c*—bone cavities. Note misaligned intercellular substance and mass of hypertrophic cells in bone structure (*black arrow*). In places with anomalies, LAGs are not visible. *Scale bar* 100 μm

For histological analysis, we randomly chose 100 individuals from the urban site (50 females and 50 males) and all specimens from the remaining two populations. Five toads from the rural site and four from the semi-urban site were rejected from age estimating due to high concentration of anomalies in entire cross section of bone tissue. The average age of populations was similar: 3.7 years at the urban and rural sites, 3.8 at the semi-urban site (Table 1). Three- and 4-year-old toads dominated in each population (Fig. 2a, b). The youngest toads were 2-year-old, the oldest—7. There were no significant differences in the age structure between populations (Kruskal–Wallis H test H = 0.47, *p* > 0.05).

Anomalies in bone tissue were observed at all studied sites, but significant differences in the frequency of their occurrence were found (Pearson chi-square test, *p* < 0.05). Detailed analysis showed differences between urban and the other two sites (*p* < 0.05) but not between semi-urban and rural sites (*p* > 0.05). In the rural population, anomalies were found in 19 % of toads; in semi-urban, in 23 %; and in urban, in 82 % (Table 1). In impaired bone tissue, we found masses cells look like hypertrophic, misaligned intercellular substance, irregular collagen fibres and outer edges of bone (Fig. 2b, d). In all cases, a full range of these changes was present. Anomalies were observed through the entire thickness of tissue, mostly in the epiphyseal part of the phalanx, but in some cases also in the diaphysis. In such deformed parts of bone, LAGs were not visible (Fig. 2b, d). In individuals without malformations, the bone structure was smooth, osteocytes were arranged concentrically in bone holes and between them homogeneous mineralized intercellular substance was visible (Fig. 2a, c). Layers of bone lied parallel to each other and LAGs were clearly visible. Nutrition canals were observed in both types of bones (Fig. 2a, b). The level of bone calcification was comparable for both normal and abnormal tissues. Importantly, we found no statistical differences in the frequency of bone abnormalities occurrence separately for males and females (GLMM, *p* > 0.05) and years of life (GLMM, *p* > 0.05).

## Discussion

### Age and sex ratio

In each population, we found only adult individuals between the ages characteristic for mating toads (Hemelaar 1986, Reading 1988, 1991, 2003). Lack of juveniles is associated with sampling of the animals during the spring migration to the breeding site, where sexually immature individuals do not appear (Socha and Ogielska 2010). Thus, it seems that skeletochronological procedure based on phalanges from killed amphibians during breeding migration is especially useful to age estimation (of the reproductive population); however, the effect of road mortality on age structure of amphibians is still unexamined.

In contrast to age structure, sex ratio of dead toads was significantly different in the most polluted area in our research (Table 1). The adult sex ratio is strong phylogenetically associations between genetic sex-determination systems and the demography (Pipoly et al. 2015). Predominantly, in the case of amphibians, males are more numerous, due partly to their arriving at the breeding place earlier and departing later than females, but also to competition between males (Höglund 1989; Loman and Madsen 2010). Recent research showed that sex ratio in amphibians can be environmentally manipulated due to estrogenic contamination or wastewater treatment work (Willingham and Crews 1999; Lange et al. 2011; Zhelev et al. 2014; Lambert et al. 2015). Male-biased adult sex ratios are natural in most members of the Anura order, but Lambert et al. (2015) have demonstrated increasing feminisation along with suburbanisation. These studies imply that endocrine disruption in suburban aquatic systems significantly alters the sex ratio in populations of metamorphosed frogs *Rana (Lithobates) clamitans*. Also, *Pelophylax ridibundus* and *Bufotes viridis* showed altered sex ratios in anthropogenically polluted biotopes (Zhelev et al. 2014). However, our observations should be extended to a series of subsequent reproductive seasons and also with use metamorph to check their sex ratio.

### Bones abnormalities

We found that the toad population from the urban site was prone to a greater extent to the formation of anomalies in bone tissue than those from the semi-urban and rural sites (Table 1). One of the factors may be the effect of the food base or hibernation site on the concentration of contaminants in the body. For instance, the common toad forage on earthworms and other invertebrates, which constitute a dangerous source of heavy metals for amphibians (Gish and Christensen 1973; Darling and Thomas 2005). Additionally, some contaminated soil or water can be absorbed in the catching and swallowing of victims (Bulog et al. 2002). On the other hand, specimens with the highest concentrations of heavy metals do not survive hibernation. As well, toads which hibernate in highly contaminated soil may be subject to locomotor impairment (James et al. 2004; Hanafy and Soltan 2007).

The histological changes that we detected may result from the use of certain elements in bone mineralisation and rebuilding. Bone tissue may accumulate high concentrations of metals such as lead (Pb), cadmium (Cd), iron (Fe), copper (Cu), nickel (Ni), zinc (Zn) (Flyaks and Borkin 2004), and thallium (Tl) (Dmowski et al. 2015). Pb, for instance, is deposited in bone on the basis of exchange with calcium (Ca) ions. This leads to a reduction in bone mineral density as well as a more porous structure and distorted collagen fibres in newly formed bone. Consequently, the risk of susceptibility to fractures, and disorders in healing increase clearly (Büsselberg 1995; Janus et al. 2007; Prutsman-Pfeiffer 2008). However, development and mineralization of periosteal bone occurs in metamorphosing tadpoles, so it is possible that, in highly contaminated ponds, newly metamorphosed toads may have deformed bone structure.

The presence of heavy metals in the environment affects the contents of Ca and phosphorus (P) in bone tissue. Simon et al. (2012) indicates that the percentage ratios of these elements at the urban pond were lower (20.5 and 14.6 %, respectively) than at the rural (30 and 22 %, respectively). The lower chemical elements concentrations lead to reduced hydroxyapatite content in bones. Although heavy metal concentration in the toads’ bone tissue was not determined in this study, it may be assumed to be the cause of abnormal bone structure in the examined toads. A similar phenomenon has been described for rats, whereby exposure to Cd and Pb led to disturbances in bone structure connected with an early stage of osteoporosis in these young individuals (Duranova et al. 2014; Lu et al. 2014). As well, studies of European pond turtles at three natural locations differing in their degree of pollution showed that nutrient-rich (nitrogen and phosphorus) and polluted waters exert a major impact on the pathogenesis of plastron and carapace including necrosis and osteoporosis (atrophy of bone tissue) (Aleksić-Kovačević et al. 2014).

Additionally, we found a relatively large proportion of individuals with abnormalities from the semi-urban and rural sites. In other agricultural site in Poland, high concentrations (11 % of all elements) of sulphur (Si) were found in the shafts of abnormal bones, but a negligible amount (0.3 %) in tissue without malformations (unpublished data). As well, low concentrations of molybdenum (Mo) and aluminium (Al) were found only in the epiphyses of impaired bones. Thus, it seems that other causes may also be responsible for these kinds of degeneration, but this requires further complex research.
